# Optimization of Ultrasonic-assisted Extraction of Fatty Acids in Seeds of *Brucea Javanica* (L.) Merr. from Different Sources and Simultaneous Analysis Using High-Performance Liquid Chromatography with Charged Aerosol Detection

**DOI:** 10.3390/molecules22060931

**Published:** 2017-06-04

**Authors:** Zhuona Wu, Ling Li, Ning Li, Tong Zhang, Yiqiong Pu, Xitong Zhang, Yue Zhang, Bing Wang

**Affiliations:** 1 Experiment Center for Teaching and Learning, Shanghai University of Traditional Chinese Medicine, No. 1200, Cailun Road, Pudong New District, Shanghai 201203, China; m18700957985@163.com (Z.W.); liling_sh@163.com (L.L.); puyiq@163.com (Y.P.); zy0217@163.com (Y.Z.); 2 School of Pharmacy, Shanghai University of Traditional Chinese Medicine, No. 1200, Cailun Road, Pudong New District, Shanghai 201203, China; 3 Institute of Chinese Materia Medica, Shanghai University of Traditional Chinese Medicine, No. 1200, Cailun Road, Pudong New District, Shanghai 201203, China; mailtolining@gmail.com; 4 Shanghai Xiangshan Hospital of Traditional Chinese Medicine, No. 528, Middle Fuxing Road, Huangpu District, Shanghai 200020, China; zhangxitong1990@126.com

**Keywords:** charged aerosol detector, chromatographic fingerprint, fatty acids, high-performance liquid chromatography, response surface methodology

## Abstract

Our research aimed to optimize the oil extraction process and determine the fatty acids in *Brucea javanica* (L.) Merr. seeds. The extraction technology was optimized using response surface methodology. A Box-Behnken design was employed to investigate the effects of three independent variables on an ultrasonic-assisted extraction technique, namely, sonication time (X_1_: 20–40 min), liquid–solid ratio (X_2_: 16:1 mL/g–24:1 mL/g), and ethanol concentration (X_3_: 90%–100%). The optimum conditions of sonication time, liquid–solid ratio, and ethanol concentration were 40 min, 24:1 mL/g, and 100%, respectively. The content of fatty acids and the oil yield were 14.64 mg/g and 16.87%, respectively, which match well with the predicted models. The optimum number of extraction times was eventually identified as two. A new rapid method for the qualitative and quantitative analysis of the fatty acids of *B. javanica* (L.) Merr. seed oil using HPLC with a charged aerosol detector was described. The fatty acid contents of 14 batches of *B. javanica* (L.) Merr. seed oil were determined, and the relevance and difference were analyzed by fingerprint analysis. The fingerprint has five common peaks, and the similarity was greater than 0.991. HPLC analysis represents a specialized and rational approach for the quality identification and comprehensive evaluation of *B. javanica* (L.) Merr. seed oils.

## 1. Introduction

*Brucea javanica* (L.) Merr. (Simaroubaceae plant), a traditional Chinese herb, is widely distributed in the southern provinces of China, such as Guangdong, Guangxi, Yunnan, and Hainan [1]. *B. javanica* (L.) Merr. seed oil (BJO) is an extract of the dried nucleoli of *B. javanica* (L.) Merr.. The primary component of BJO is fatty acid (FA), which includes oleic, linoleic, linolenic, palmitic, and stearic acids. Oleic and linoleic acids are the main active ingredients in BJO [2], which possesses various biological and pharmacological activities, and is commonly used to treat various cancers, such as lung, gastrointestinal, and liver cancers [3]. Its mechanisms of the tumor growth inhibition include inhibition of DNA synthesis, suppression of tumor multidrug resistance, and destruction of tumor cell membrane systems [4].

As is well known, the study of extraction methods is very important, and various techniques for the extraction of BJO have been developed, including supercritical fluid extraction, soakage extraction and ultrasonic-assisted extraction (UAE). It is reported that UAE can achieve better extractions of natural products [5,6]. The effect of ultrasonic waves is strong, as they can destroy the cells of plant, which is especially suitable for seeds [7,8]. UAE is seen as an ideal option for the edible oil industry because of improvements in efficiency and speed and because it can be performed at low operation temperatures which avoids thermal damage to the extracts and preserves the structural and molecular properties of bioactive compounds. Many factors affect the extraction efficiency of UAE. Some of these are ultrasonic power, extraction time, extraction temperature, and solvent to solid ratio [9].

Response surface methodology (RSM) is an efficient mathematical and statistical technique for optimizing complex extraction procedures [10,11]. Box-Behnken design (BBD) is a RSM design method which is widely applied to different classes of compounds, including phenolics [12], polysaccharides [13], FAs [7], and flavonoid compounds [14]. 

The most commonly used methods for the analysis of FA include gas chromatography [15,16,17] and high-performance liquid chromatography (HPLC), typically connected with an ultraviolet (UV), evaporative light-scattering, diode array, and/or mass spectrometry detector [18,19,20]. Most FAs lack or have weak UV absorptions, and therefore, they must be derivatized before determination [21,22,23]. In 2003, a new alternative detector, the charged aerosol detector (CAD), was introduced [24]. CAD is a mass sensitive and universal detector suitable for the routine determination of many non-volatile or weak-volatile chemical species and other compounds that contain weak chromophores [25]. This detector can analyze triacylglycerols, lipids, and other substances [26,27,28] and it can also directly detect FA without derivatization [29,30].

The construction of a chromatographic fingerprint plays an important role in the quality control of complex herbal medicines [31]. This technique emphasizes the systemic characterization of sample compositions and focuses on identifying and assessing the stability of plants. The identity, stability, and consistency of Traditional Chinese Medicines (TCMs), as well as the identification of adulterants, can be determined by their chromatographic fingerprints [32].

The current study aimed to adequately extract BJO with RSM and establish a sensitive and selective HPLC-CAD method (for the simultaneous determination of five FAs and to develop a characteristic fingerprint to control the quality of the raw herb. This study focuses on the optimization of a complete set of extraction and analysis methods of BJO and the establishment of the chromatographic fingerprint of TCM to provide a reliable basis for the further study of *B. javanica* seeds.

## 2. Results and Discussion 

### 2.1. Validation of HPLC-CAD Method

#### 2.1.1. Calibration Curves, Limits of Detection, and Quantification

**Figure 1:**
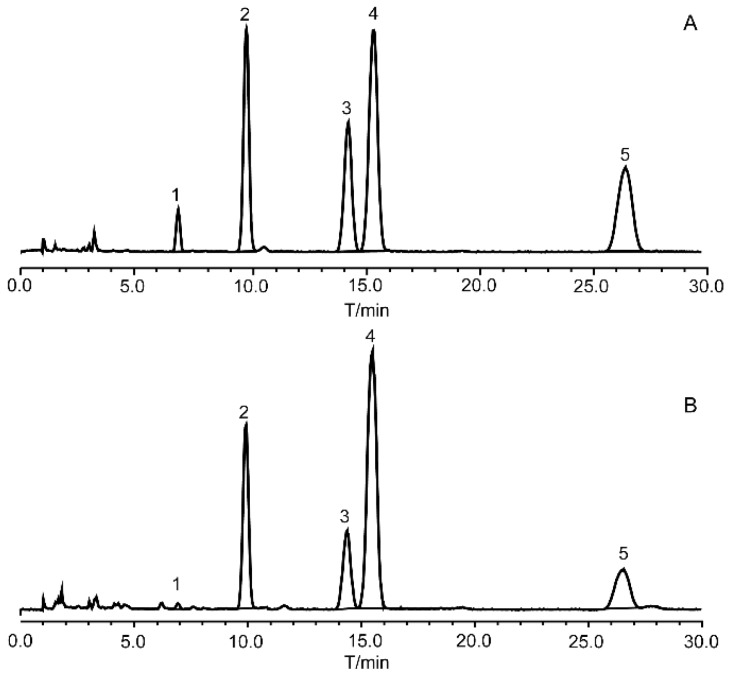
HPLC chromatograms of mixed standard solution **(A)** and sample solution **(B)** (1—linolenic acid, 2—linoleic acid, 3—palmitic acid, 4—oleic acid, and 5—stearic acid).

The regression equations of the five FAs listed in Table 1 were linear with the correlation coefficients (R^2^) and were between 0.9983 and 0.9999. The lowest LOD and LOQ were obtained with linoleic acid (0.368 μg/mL and 2.624 μg/mL, respectively), whereas the highest LOD and LOQ were obtained with stearic acid (1.958 μg/mL and 6.800 μg/mL, respectively). The chromatograms of mixed standard solution and sample solution are shown in Figure 1.

#### 2.1.2. Precision

Table 2 shows the precision. Relative standard deviation (RSD) was used to express the precision. The RSD values of intra-day and inter-day precision for the peak area were lower than 3%, which indicates that this method is stable.

#### 2.1.3. Accuracy

The results of the recovery experiment (Table 3) showed that the overall average recoveries were 94.88% to 105.00%, which indicated that the current method is robust and suitable for the determination of FAs in BJO.

### 2.2. Effect of Independent Variables on the Content of FAs and the Oil Yield

**Figure 2:**
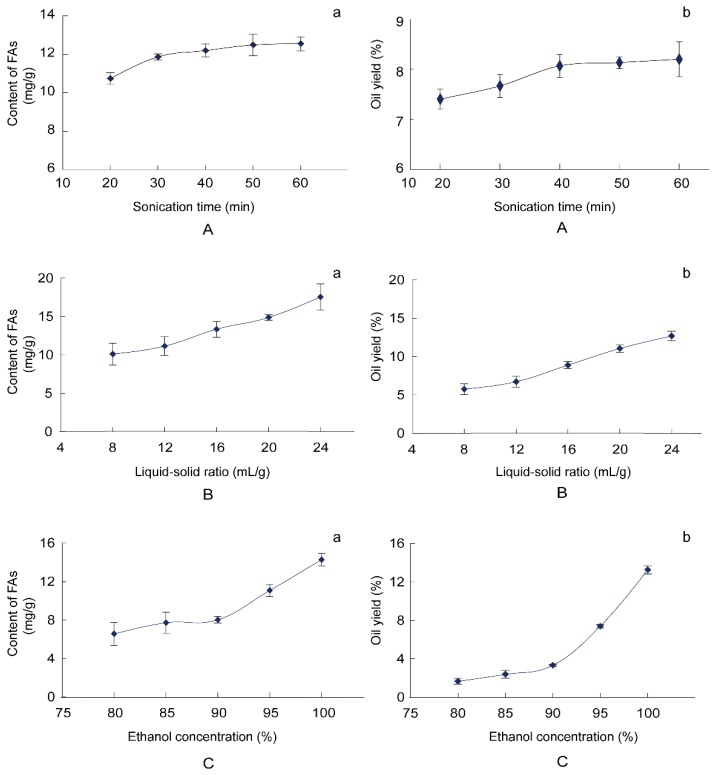
Effects of different factors on content of FAs and oil yield. The effects of sonication time (min), liquid-solid ratio (mL/g), and ethanol concentration (%) are shown in **A**, **B**, and **C**, respectively. The content of FAs (mg/g) and the oil yield (%) are shown in a and b, respectively. Data are shown as mean ± SD (*n* = 3).

The content of FAs and the oil yield of BJO affected by different sonication time (20–60 min) are presented in Figure 2A, where two other factors, liquid–solid ratio and ethanol concentration, were fixed at 12:1 mL/g and 95%, respectively. The content of FAs and the oil yield of BJO increased during the initial 40 min and then slowed down until they reached an equilibrium. The content of FAs and the oil yield of BJO affected by the different liquid–solid ratio (8:1 mL/g–24:1 mL/g) are seen in Figure 2B, where two other factors, sonication time and ethanol concentration, were fixed at 30 min and 95%, respectively. The liquid–solid ratio significantly affected the content of FAs and the oil yield. The content of FAs and the oil yield of BJO increased rapidly with the liquid-solid ratio increased from 8:1 mL/g to 24:1 mL/g, and reached the maximum value at 24:1 mL/g. The content of FAs and the oil yield of BJO affected by different ethanol concentration (80–100%) are shown in Figure 2C, where two other factors, sonication time and liquid–solid ratio, were fixed at 30 min and 12:1 mL/g, respectively. The content of FAs and the oil yield of BJO were little when ethanol concentration was lower than 90%. However, the content of FAs and the oil yield increased rapidly when ethanol concentration changed from 90% to 100% and reached the peak value at 100% concentration.

Combining with the results above and considering the time and cost savings, this study took sonication times of 20, 30, and 40 min; liquid-solid ratios of 16:1, 20:1, and 24:1 mL/g; and ethanol concentrations of 90%, 95%, and 100% for further study objects in the BBD experiment.

### 2.3. Model Fitting

Analysis of variance (ANOVA) for the model is presented in Table 4. The results indicated that the model used to fit response variable was significant (*p* < 0.05, *p* < 0.01) and adequate to represent the relationship between the response and the independent variables. The coefficient (R^2^) of the content of FAs and the oil yield were 0.8650 and 0.9456, respectively. The calculated models had no significant lack of fit at *p* > 0.05, which suggests a good fit. The predicted models reasonably represented the observed values. The content of FAs and the oil yield were affected most significantly by ethanol concentration (X_3_) (*p* < 0.01, *p* < 0.0001), followed by liquid-solid ratio (X_2_) (*p* < 0.05, *p* < 0.01), and sonication time (X_1_) (*p* = 0.2669, *p* = 0.1212). The content of FAs shows that the quadratic parameters (X_1_^2^, X_3_^2^) were significant (*p* < 0.05, *p* < 0.01), whereas the quadratic parameters (X_2_^2^) and all the interaction parameters were insignificant (*p* > 0.05). In the oil yield, all the interaction parameters (X_1_X_2_, X_1_X_3_, and X_2_X_3_) and the quadratic parameters (X_1_^2^, X_2_^2^, and X_3_^2^) were insignificant (*p* > 0.05). The predicted response could be expressed by the following second-order polynomial equations in terms of coded values:

$$Y_{1}=10.72+0.36X_{1}+0.7683X_{2}+1.16X_{3}+0.47X_{1}X_{2}+0.11X_{1}X_{3}+0.42X_{2}X_{3}+1.04{X_{1}}^{2}+0.32{X_{2}}^{2}-1.49{X_{3}}^{2}$$

$$Y_{2}=8.88+0.64X_{1}+1.53X_{2}+4.29X_{3}+0.88X_{1}X_{2}+0.31X_{1}X_{3}+0.73X_{2}X_{3}+\;0.83{X_{1}}^{2}+0.21{X_{2}}^{2}-0.44{X_{3}}^{2},$$

where Y_1_ and Y_2_ are the FA contents and the yield, respectively, and X_1_, X_2_, and X_3_ are the coded variables for ultrasonic time, liquid–solid ratio, and ethanol concentration, respectively.

### 2.4. Analysis of Response Surface

**Figure 3:**
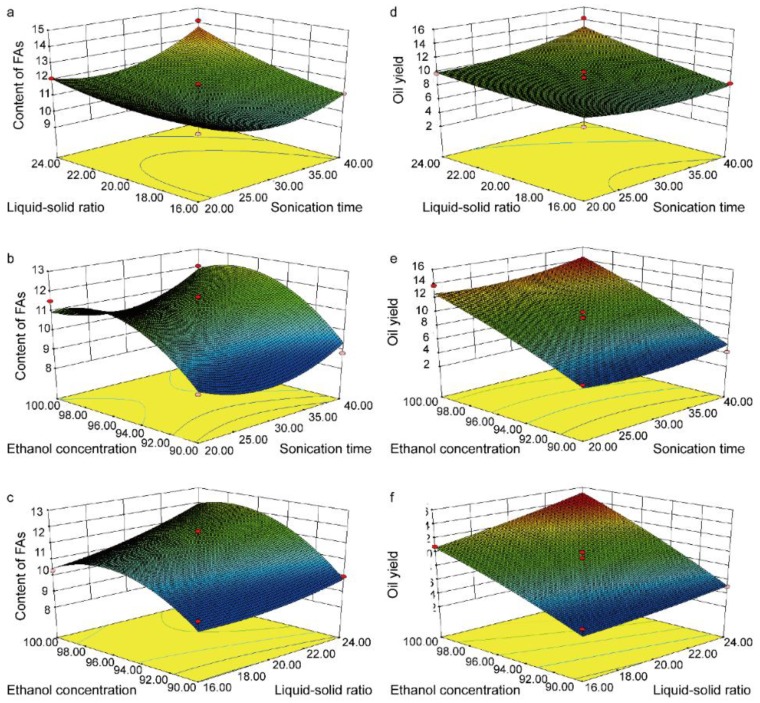
Response surface plot for interactions between three independent variables on contents FAs (mg/g, **a**, **b**, and **c**) and oil yield (%, **d**, **e**, and **f**). Two variables were plotted against each other in each panel.

**Figure 4:**
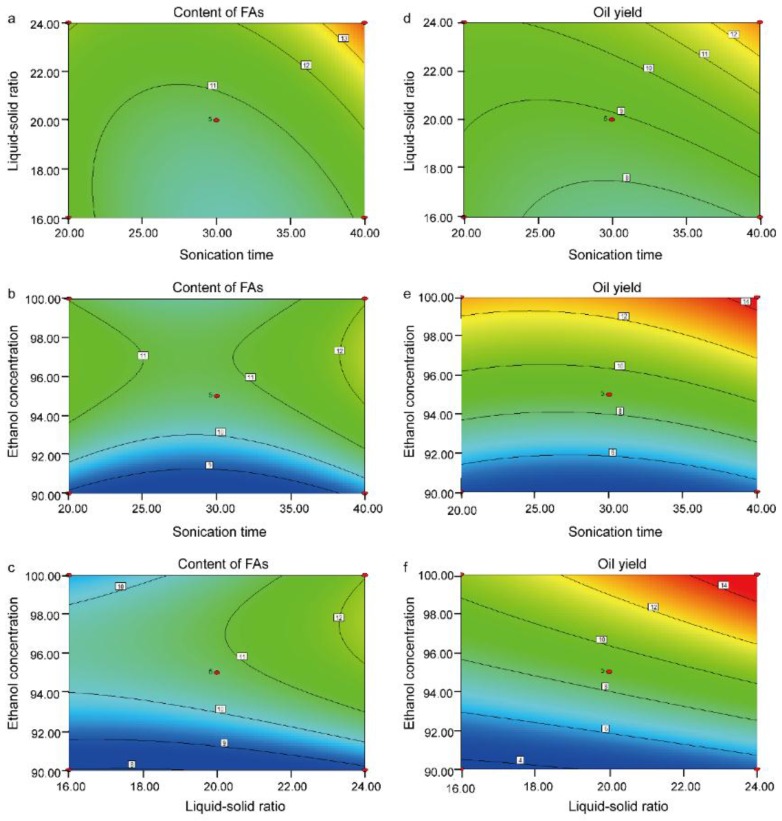
Contour plot for interactions between three independent variables on content of FAs (mg/g, **a**, **b**, and **c**) and oil yield (%, **d**, **e**, and **f**). Two variables were plotted against each other in each panel.

The 3D response surface plots were obtained by varying two variables within the experimental range under investigation and holding another variable at its “0” level. Figure 3 and Figure 4 show the 3D response surface plots and 2D contours for the optimization conditions of ultrasonic extraction of BJO, respectively. The effects of sonication time (X_1_) and liquid–solid ratio (X_2_) on the content of FAs and the oil yield are shown in Figure 3a,d, respectively. The response values were increased with increases in sonication time (X_1_) and liquid–solid ratio (X_2_). Figure 3b,e show the effect of sonication time (X_1_) and ethanol concentration (X_3_) on the content of FAs and the oil yield, respectively. The response values increased with the increases in sonication time (X_1_). Further increase in ethanol concentration (X_3_) resulted in the enhancement of this trend. Figure 3c,f show the effect of liquid–solid ratio (X_2_) and ethanol concentration (X_3_) on the content of FAs and the oil yield, respectively. Both increased with the increases in liquid–solid ratio (X_2_) and ethanol concentration (X_3_).

### 2.5. Optimization of Extraction Parameters and Validation of the Model

The optimized results showed that the maximum content of FAs and oil yield by UAE could achieve 13.94 mg/g and 17.86%, respectively. The software predicted the optimum sonication time, liquid–solid ratio, and ethanol concentration to be 40 min, 24:1 mL/g, and 100%, respectively. Three parallel experiments were carried out under the optimal conditions. The actual values of the content of FAs and the oil yield were 14.64 mg/g and 16.87%, respectively, which were close to the predicted values. This result indicated that the optimization in the present study is reliable.

### 2.6. Effect of Extraction Times on Content of FAs and Oil Yield

The content of FAs and the oil yield of BJO became higher with the increase of extraction time. In the first extraction, the content of FAs and the oil yield of BJO were the highest (14.64 mg/g, 16.87%), and the content of FAs and the oil yield of the two instances that followed decreased quickly (The content of FAs and the oil yield obtained by second times were 2.81 mg/g and 5.7%, respectively, and the third times were 0.67 mg/g and 1.2%, respectively.). The relative cumulative value of two-time extraction has reached 95%, indicating that two-time extractions of BJO have been completed. The oil yield extracted twice was 22.57%, which was slightly higher than that of Ge et al. (21.35%) [33]. The two times were selected, taking into account the cost of extraction.

### 2.7. Comparison of Content of FAs

The content of FAs of BJO from different sources are shown in Table 5. The main components of BJO were oleic acid (16.105–77.477 mg/g) and linoleic acid (4.588–27.270 mg/g), followed by palmitic acid (3.242–12.936 mg/g) and stearic acid (1.469–7.497 mg/g), whereas the content of linolenic acid (less than 0.178 mg/g) was limited. The trend was consistent with reported studies [33,34]. Linolenic acid was not discovered in both works. However, linolenic acid was detected by the CAD detector in our study, which shows that CAD has high sensitivity. In the study of Ge et al. [33], BJO (collected from Guangxi) was extracted with petroleum ether by Soxhlet extraction. Their experimental data showed that the content of oleic acid was 67.45%, higher than our experimental results (Guangxi-1: 77.48 mg/g (46.43%); Guangxi-2: 69.68 mg/g (43.97%); Guangxi-3: 50.38 mg/g (36.91%)). The content of linoleic acid was 18.92%, slightly higher than our data (Guangxi-1: 25.49 mg/g (15.27%); Guangxi-2: 27.27 mg/g (17.21%); Guangxi-3: 22.28 mg/g (16.32%)). The content of stearic acid showed slight difference (Ge et al.: 4.93% [33]; Guangxi-1: 6.88 mg/g (4.12%); Guangxi-2: 6.89 mg/g (4.34%); Guangxi-3: 6.06 mg/g (4.41%)). However, the content of palmitic acid in our research (Guangxi-1: 12.80 mg/g (7.67%); Guangxi-2: 12.94 mg/g (8.16%); Guangxi-3: 11.18 mg/g (8.19%) is higher than that of Ge et al. (7.04%) [33]. One reason for the lower content of FAs in our research may be that the sample is affected by the harvesting time and region. Another reason may be that the efficiency of Soxhlet extraction with petroleum ether is better than that of ultrasonic-assisted extraction with ethanol. For the contents of oleic acid and linoleic acid, Guangdong-4, Guangxi-1, and Guangxi-2 were higher, while Guangdong-1, Fujian-1, Hebei, and Yunnan were relatively lower. For the total content of FAs, Guangdong-4, Guangxi-1, and Guangxi-2 were higher, followed by Guangxi-3, Guangdong-5, and Guangdong-6, while Guangdong-1, Fujian-1, Hebei, and Yunnan were relatively lower. 

When considering extraction of oleic acid as the main active substance of BJO, the herbal sources from Guangxi and Guangdong are the better alternatives. However, in order to obtain higher total contents of FAs, the herbs from Guangxi and Guangdong should be chosen as the preferred sources instead of those from Fujian, Hainan and Yunnan. The experimental results also indicated that the varieties and contents of FAs in different sources were significantly different. Due to the planting area, harvest time, climate, and other factors, even if the herbs came from the same sources, their content of FAs of BJO were also somewhat different.

### 2.8. HPLC Fingerprint, Cluster, and Principal Component Analysis

**Figure 5:**
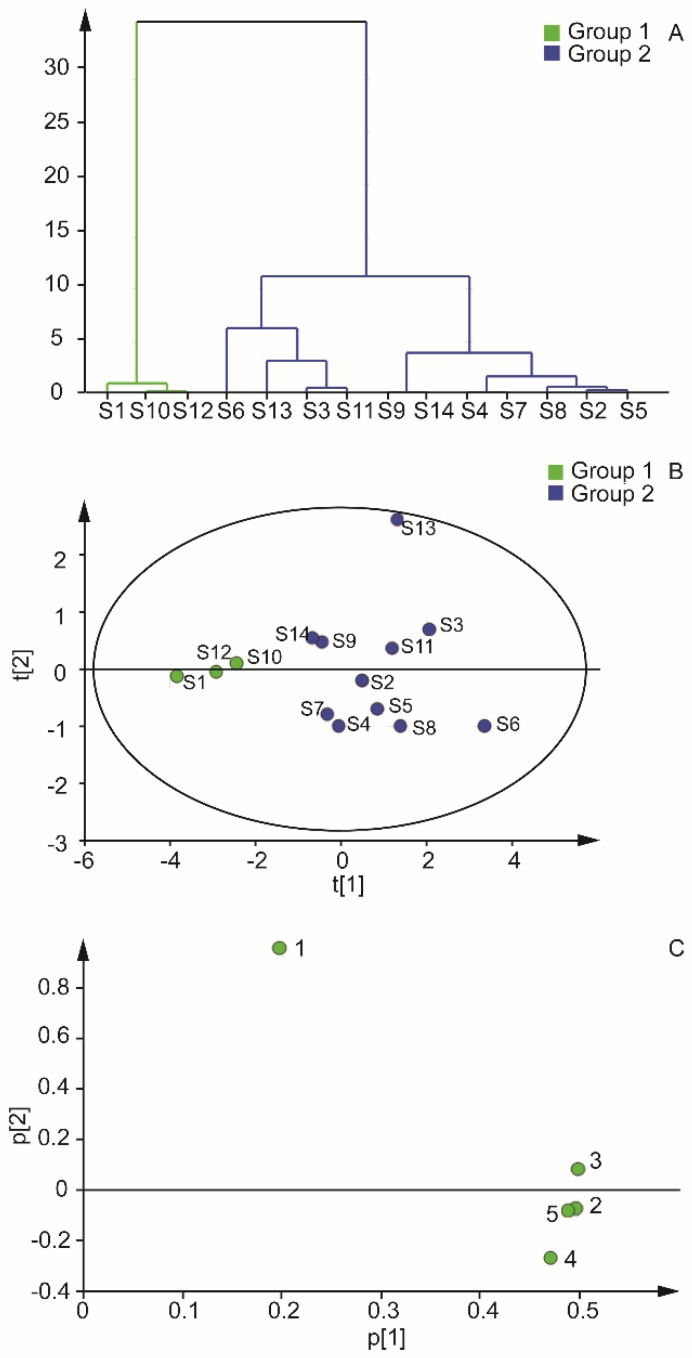
Cluster analysis and principal components of 14 samples. (**A**, **B**, and **C** are dendrogram, score plot, and loading scatter plot, respectively.)

The chromatogram of sample 1 (S1) was set as the reference map, and the fingerprint of the FA part of BJO of 14 batches was established. The control fingerprint was automatically generated by the system, and five common peaks were obtained. The similarity of all samples was higher than 0.991, which indicates that the FA composition of BJO of different regions had good similarity. The cluster analysis and principal component analysis were performed with the peak area of five common peaks of FA. Figure 5A,B show that the 14 samples could be clustered into two categories. Samples 1, 10, and 12 were combined into one class, and the rest of the samples were a another category. The contribution rate of the first and second principal components were 78.9% and 18.8%, respectively. The results were affected by linoleic, oleic, palmitic, and stearic acids (Figure 5C).

## 3. Materials and Methods

### 3.1. Materials and Standards

Ethanol, acetonitrile, formic acid, and petroleum ether (60 °C to 90 °C) were obtained from Sinopharm Chemical Reagent Co., Ltd., Shanghai, China. Deionized water was purchased from Wahaha Co., Ltd., Hangzhou, China. Five FA standards (i.e., linolenic acid, linoleic acid, palmitic acid, oleic acid, and stearic acid) were purchased from Sigma (St. Louis, MO, USA). The raw *B. javanica* seeds were collected from different regions in China (Guangdong, Guangxi, Fujian, Hebei, Yunnan, and Hainan). All samples were authenticated by Dr. Hongmei Zhang of School of Pharmacy, Shanghai University of Traditional Chinese Medicine and stored at the Experiment Center for Teaching and Learning, Shanghai University of Traditional Chinese Medicine.

### 3.2. Extraction of Oil

The dried seeds were ground (60-mesh) with a mill (FWJ-03, Minye Industrial and Trading Co., Shanghai, China). The powder (5 g) was placed into a conical flask (250 mL). Ethanol (120 mL) was added and then the flask was placed in an ultrasonic cleaning bath (SB5200D, 40 kHz, Ningbo Scientz Biotechnology Co., Ltd., Zhejiang, China) for 40 min. The filtrate was collected, concentrated, and extracted with petroleum ether. BJO was obtained after solvent evaporation.

### 3.3. Preparation of Sample Solution

The BJO of different origins was extracted according to the optimal condition of the above extraction experiments. Appropriate BJO was weighed and dissolved in acetonitrile. The sample was swirled for 2 min, then sonicated for 2 min to disperse the contents evenly, and finally filtered through a 0.45 μm membrane.

### 3.4. HPLC-CAD Analysis

Measurements were carried out using HPLC U3000 with a Corona^®^ charged aerosol detector (Thermo Scientific, Idstein, Germany). A XB-C18 (150 mm × 4.6 mm, 5 μm) column (Welch, Shanghai, China) was used for sample separation at 30 °C. The injection volume was 10 μL. Isocratic elution (15:85) was applied using 0.05% (*v*/*v*) formic acid in water as mobile phase A and 0.05% (*v*/*v*) formic acid in acetonitrile as mobile phase B. The flow rate was set to 1 mL/min. The settings for the CAD were as follows: gas, nitrogen; pressure, 35 psi; filter, none; collection frequency, 2 Hz; and acquisition range, 100 pA.

### 3.5. Validation of HPLC-CAD Method

#### 3.5.1. Calibration Curves, Limits of Detection, and Quantification

Linolenic acid, linoleic acid, palmitic acid, oleic acid, and stearic acid were accurately weighed and dissolved in acetonitrile to make stock solutions. The stock solutions were diluted to five appropriate concentrations and analyzed in triplicate to construct the calibration curves. The limits of detection (LOD) and the limits of quantification (LOQ) of each analyte were calculated on the peak response at signal-to-noise of 3 and 10, respectively.

#### 3.5.2. Precision

The precision of the method was evaluated by intra-day and inter-day precision. Three samples concentrations were analyzed six times daily to determine the intra-day precision. Three samples were analyzed three times on three consecutive days to test the inter-day precision.

#### 3.5.3. Accuracy

The accuracy of this method was evaluated by the recovery test. Experiments were conducted by adding three different concentrations (50, 100, and 150 mg/mL) of five standard compounds. Each set of samples was repeated three times, and the average recovery rate of each compound was calculated. The equation for calculating the accuracy of HPLC method was as follows:

Recovery (%) = (detected amount－original amount) / added amount × 100%

### 3.6. Content of FAs and Oil Yield Determination

The efficiency of the UAE was evaluated using the content of FAs and the oil yield as indexes. The content of FAs were equal to the sum of the content of linolenic, linoleic, palmitic, oleic, and stearic acids. The oil yield was calculated as follows:

$$\mathrm{Yield}\;\left(\%\right)=\frac{W_{0}}{W_{S}}\times 100\%$$

W_0_ and W_S_ are the weight of the oil extracted from the sample (g) and the weight of the sample (g), respectively. 

### 3.7. Optimization of UAE

The extraction conditions were determined first by a single-factor test, and the effects of independent variables and their interactions on response variables were evaluated by BBD. The optimal extraction conditions that achieve the highest extraction rate and the highest FA contents were predicted by establishing a mathematical model. RSM optimized the extraction parameters. A three-factor, three-level BBD was applied to determine the optimal conditions for the UAE of BJO. Table 6 shows the range and center point values of the three independent variables based on the results of the single-factor test. The sonication time (min, X_1_), liquid-solid ratio (mL/g, X_2_), and ethanol concentration (%, X_3_) were chosen as independent variables. 

The content of FAs and the oil yield were selected as the responses for the combination of the independent variables. The design of experiments are presented in Table 7. Experimental runs were randomized to minimize the effects of unexpected variability in the observed responses.

Experimental data were fitted to a quadratic polynomial model and the obtained regression coefficient. The nonlinear computer-generated quadratic model used in the response surface was as follows:

$$Y=\beta_{0}+\sum_{i=1}^{3}\beta_{i}X_{i}+\sum_{i=1}^{3}\beta_{\mathrm{ii}}{X_{i}}^{2}+\sum_{i\neq j=1}^{3}\beta_{\mathrm{ij}}X_{i}X_{j},$$

where Y, β_0_, β_i_, β_ii_, and β_ij_ indicated the predicted response, the intercept term, the linear coefficient, the squared coefficient, and the interaction coefficient, respectively.

### 3.8. Determination of Extraction Times

Extraction was performed under optimum conditions. Three samples were taken, and each sample was extracted three times. The content of FAs and the oil yield were calculated.

### 3.9. Data Analysis

The response obtained from each set of experimental design was assessed by the Design Expert software (Trial Version 8.0.5b, Stat-Ease Inc., Minneapolis, MN, USA). The determination of fingerprints was carried out using the Similarity Evaluation System for Chromatografic Figureprint of Traditional Chinese Medicine software (Version 2012, Chinese Pharmacopoeia Commission, Beijing, China). Cluster analysis and principal component analysis were evaluated by Simca (Version 13.0, Umetrics AB, Umea, Sweden).

## 4. Conclusions

The present study developed and applied a highly efficient extraction method to extract BJO. The main variables affecting the content of FAs and oil yield were optimized by RSM based on a BBD. The proposed method could provide favorable extraction efficiency under optimal conditions. Ultrasonic-assisted extraction times were investigated in order to achieve the higher extraction rate of BJO. Moreover, a rapid, sensitive, and reliable method for the quantitative analysis of FAs in *B. javanica* (L.) Merr. seeds was developed. The analytical method used in this study avoided the complicated pretreatment process of samples and showed good sensitivity and low detection limit. The method was successfully applied to the simultaneous determination of five kinds of FAs in 14 batches of *B. javanica* (L.) Merr. seeds from different regions in China. The analysis results showed that *B. javanica* (L.) Merr. seeds is rich in oleic and linoleic acids, and the content of FAs varied significantly in *B. javanica* (L.) Merr. seeds from different regions. Moreover, the results indicated that the proposed method was sufficiently competent to determine FAs in various natural medicines and TCM and demonstrates great potential to analyze formulations and products containing BJO.

## Figures and Tables

**Table 1:** Linearity, LOD, and LOQ of the investigated compounds.

Compound	Retention Time (min)	Linear Range (mg/mL)	Equation	R^2^	LOD (μg/mL)	LOQ (μg/mL)
Linolenic acid	6.95	0.0012–0.0349	y = 63.533x + 0.0164	0.9999	0.504	1.080
Linoleic acid	9.95	0.0109–0.5452	y = 44.152x + 0.8404	0.9983	0.368	2.624
Palmitic acid	14.45	0.0084–0.4204	y = 53.760x + 0.2189	0.9996	1.400	4.032
Oleic acid	15.56	0.0161–0.8032	y = 46.574x + 1.3419	0.9983	1.376	3.856
Stearic acid	26.65	0.0068–0.2030	y = 81.835x + 0.0361	0.9996	1.958	6.800

**Table 2:** Intra-day and inter-day precision of standard solutions.

Compound	Concentration (mg/mL)	Intra-day (*n* = 6)	Inter-day (*n* = 3)
		RSD (%)	RSD (%)
Linolenic acid	0.0012	1.80	1.19
	0.0116	1.07	1.36
	0.0349	1.71	1.99
Linoleic acid	0.0112	1.82	2.17
	0.1118	0.37	1.42
	0.3353	1.04	2.29
Palmitic acid	0.0088	1.33	1.21
	0.0877	0.48	1.94
	0.2630	1.36	2.02
Oleic acid	0.0231	1.97	2.06
	0.2310	0.26	1.44
	0.6931	1.00	2.05
Stearic acid	0.0066	1.48	2.71
	0.0664	0.44	2.86
	0.1992	0.98	2.64

**Table 3:** Recovery of Five FAs in Samples.

Compound	Initial(mg)	Amount(mg)	Add(mg)	Found ± SD(mg)	Recovery (%)	RSD (%, *n* = 3)
Linolenic acid	8.87	0.0177	0.0089	0.0265 ± 0.0006	98.30	2.79
	8.96	0.0179	0.0090	0.0272 ± 0.0002	103.68	
	8.71	0.0174	0.0087	0.0264 ± 0.0003	102.55	
	8.48	0.0170	0.0170	0.0331 ± 0.0005	95.19	2.17
	8.71	0.0174	0.0174	0.0346 ± 0.0005	98.65	
	8.63	0.0173	0.0173	0.0336 ± 0.0006	94.91	
	8.11	0.0162	0.0243	0.0398 ± 0.0006	96.71	2.20
	8.04	0.0161	0.0241	0.0404 ± 0.0004	101.03	
	8.23	0.0165	0.0247	0.0408 ± 0.0004	98.46	
Linoleic acid	8.87	1.2306	0.6153	1.8451 ± 0.0116	99.87	2.66
	8.96	1.2431	0.6216	1.8414 ± 0.0037	96.25	
	8.71	1.2084	0.6042	1.7817 ± 0.0133	94.88	
	8.48	1.1765	1.1765	2.3642 ± 0.0061	100.95	2.21
	8.71	1.2084	1.2084	2.4752 ± 0.0070	104.83	
	8.63	1.1973	1.1973	2.4545 ± 0.0041	105.00	
	8.11	11252	1.6878	2.8308 ± 0.0307	101.06	1.84
	8.04	1.1155	1.6732	2.8386 ± 0.0064	102.98	
	8.23	1.1418	1.7127	2.9375 ± 0.1431	104.84	
Palmitic acid	8.87	0.6905	0.3453	1.0414 ± 0.0146	101.63	1.49
	8.96	0.6975	0.3478	1.0604 ± 0.0014	104.03	
	8.71	0.6781	0.3390	1.0212 ± 0.0005	101.21	
	8.48	0.6602	0.6602	1.2946 ± 0.0044	96.10	1.82
	8.71	0.6781	0.6781	1.3456 ± 0.0027	98.44	
	8.63	0.6718	0.6718	1.3411 ± 0.0080	99.62	
	8.11	0.6314	0.9470	1.6031 ± 0.0235	102.60	0.94
	8.04	0.6259	0.9389	1.6065 ± 0.0033	104.44	
	8.23	0.6407	0.9611	1.6301 ±0.0197	102.94	
Oleic acid	8.87	2.9146	1.4573	4.3238 ± 0.0055	96.70	2.71
	8.96	2,9442	1.4721	4.4206 ± 0.0068	100.29	
	8.71	2.8620	1.4310	4.3215 ± 0.0102	101.99	
	8.48	2.7864	2.7864	5.4940 ± 0.0036	97.17	1.15
	8.71	2.8620	2.8620	5.6369 ± 0.0138	96.96	
	8.63	2.8357	2.8357	5.5337 ± 0.0057	95.14	
	8.11	2.6649	3.9973	6.6010 ± 0.0695	98.47	1.29
	8.04	2.6419	3.9628	6.6026 ± 0.0303	99.95	
	8.23	2.7043	4.0564	6.8029 ± 0.3343	101.04	
Stearic acid	8.87	0.3869	0.1930	0.5709 ± 0.0018	95.80	2.94
	8.96	0.3899	0.1950	0.5799 ± 0.0020	97.42	
	8.71	0.3791	0.1896	0.5713 ± 0.0036	101.41	
	8.48	0.3690	0.3690	0.7205 ± 0.0037	95.24	2.33
	8.71	0.3791	0.3791	0.7566 ± 0.0038	99.60	
	8.63	0.3756	0.3756	0.7376 ± 0.0109	96.38	
	8.11	0.3529	0.5294	0.8560 ± 0.0113	95.03	1.19
	8.04	0.3499	0.5249	0.8498 ± 0.0070	95.24	
	8.23	0.3582	0.5373	0.8799 ± 0.0062	97.10	

**Table 4:** ANOVA for the regression equation.

Source	Content of FAs (mg/g)					Oil Yield (%)				
	SS	DF	MS	F	P	SS	DF	MS	F	P
Model	32.63	9	3.63	4.98	0.0229	178.73	9	19.86	13.52	0.0012
X_1_	1.06	1	1.06	1.46	0.2669	3.24	1	3.24	2.20	0.1812
X_2_	5.53	1	5.53	7.60	0.0282	18.79	1	18.79	12.79	0.009
X_3_	10.76	1	10.76	14.80	0.0063	147.32	1	147.32	100.27	<0.0001
X_1_X_2_	0.89	1	0.89	1.23	0.3045	3.06	1	3.06	2.08	0.192
X_1_X_3_	0.05	1	0.05	0.07	0.8039	0.39	1	0.39	0.27	0.622
X_2_X_3_	0.69	1	0.69	0.95	0.3629	2.13	1	2.13	1.45	0.2675
X_1_^2^	4.57	1	4.57	6.29	0.0405	2.92	1	2.92	1.99	0.2012
X_2_^2^	0.44	1	0.44	0.60	0.4635	0.18	1	0.18	0.12	0.7378
X_3_^2^	9.35	1	9.35	12.86	0.0089	0.80	1	0.80	0.55	0.4837
Residual	5.09	7	0.73			10.28	7	1.47		
Lack of Fit	1.65	3	0.55	0.64	0.6278	8.06	3	2.69	4.84	0.0808
Pure Error	3.44	4	0.86			2.22	4	0.56		
Cor Total	37.72	16				189.01	16			
R^2^	0.8650							0.9456		

X_1_, X_2_ and X_3_ represent sonication time, liquid–solid ratio and ethanol concentration, respectively; SS, DF, MS and CV represent sum of squares, degree of freedom, mean square, coefficient of variation, respectively.

**Table 5:** FA contents of BJO of different origin.

Number	Origin	Linolenic Acid (mg/g)	Linoleic Acid (mg/g)	Palmitic Acid (mg/g)	Oleic Acid (mg/g)	Stearic Acid (mg/g)	Total Contents of FAs (mg/g)
S1	Guangdong-1	0.005 ± 0.001 ^a^	4.588 ± 0.132	3.242 ± 0.138	16.105 ± 0.489	1.469 ± 0.066	25.409
S2	Guangdong-2	0.092 ± 0.004	13.254 ± 0.101	6.805 ± 0.117	34.278 ± 1.213	3.817 ± 0.048	58.246
S3	Guangdong-3	0.178 ± 0.009	14.064 ± 0.082	6.977 ± 0.209	30.492 ± 0.717	3.950 ± 0.078	55.661
S4	Guangdong-4	0.012 ± 0.001	21.593 ± 0.156	11.584 ± 0.124	65.924 ± 2.023	7.497 ± 0.129	106.610
S5	Guangdong-5	0.064 ± 0.003	17.259 ± 0.984	8.735 ± 0.274	49.433 ± 2.123	4.169 ± 0.140	79.660
S6	Guangdong-6	0.058 ± 0.003	20.321 ± 0.331	9.755 ± 0.227	44.498 ± 0.564	6.095 ± 0.100	80.727
S7	Guangxi-1	0.054 ± 0.003	25.486 ± 0.902	12.803 ± 0.473	77.477 ± 3.706	6.876 ± 0.164	122.696
S8	Guangxi-2	0.056 ± 0.001	27.270 ± 0.693	12.936 ± 0.219	69.677 ± 0.772	6.885 ± 3.840	116.824
S9	Guangxi-3	0.280 ± 0.004	22.283 ± 0.568	11.176 ± 0.209	50.379 ± 2.704	6.060 ± 0.204	90.178
S10	Fujian-1	0.070 ± 0.002	8.284 ± 0.052	4.660 ± 0.028	22.655 ± 0.054	1.646 ± 0.033	37.315
S11	Fujian-2	0.165 ± 0.006	16.404 ± 0.170	8.522 ± 0.136	36.787 ± 0.378	4.580 ± 0.069	66.458
S12	Hebei	0.056 ± 0.002	8.623 ± 0.086	4.020 ± 0.011	24.894 ± 0.227	1.907 ± 0.034	39.500
S13	Yunnan	0.255 ± 0.008	9.007 ± 0.078	5.297 ± 0.153	20.355 ± 0.578	2.441 ± 0.081	37.355
S14	Hainan	0.150 ± 0.004	11.178 ± 0.010	6.398 ± 0.088	30.067 ± 0.537	2.646 ± 0.076	50.439

^a^ Data are expressed as mean value ± SD.

**Table 6:** Independent variables and the coded and actual values used for optimization.

Independent Variable	Units	Symbol	Coded Levels		
			−1	0	1
Sonication time	min	X_1_	20	30	40
Liquid-solid ratio	mL/g	X_2_	16:1	20:1	24:1
Ethanol concentration	%	X_3_	90	95	100

**Table 7:** BBD experimental design with the independent variables and experimental data for the responses.

Run	Factor 1 (X_1_)	Factor 2 (X_2_)	Factor 3 (X_3_)	Response 1 (Y_1_)	Response 2 (Y_2_)
	Sonication Time (min)	Liquid-solid Ratio (mL/g)	Ethanol Concentration (%)	Content of FAs (mg/g)	Oil Yield (%)
1	−1	0	−1	8.71	5.00
2	0	0	0	9.81	8.33
3	0	1	1	11.40	14.25
4	1	1	0	14.16	14.25
5	0	0	0	11.73	9.17
6	−1	1	0	12.08	9.75
7	0	−1	1	9.32	10.83
8	1	0	1	12.05	14.17
9	0	−1	−1	8.53	4.50
10	0	0	0	11.70	10.00
11	1	0	−1	8.81	4.17
12	0	1	−1	8.95	5.00
13	−1	0	1	11.51	13.75
14	0	0	0	10.31	8.13
15	0	0	0	10.04	8.75
16	1	−1	0	11.14	8.33
17	−1	−1	0	10.95	7.33

## Abbreviations

Non-standard abbreviations:

ANOVA	Analysis of variance
BBD	Box-Behnken design
BJO	*Brucea javanica* (L.) Merr. seed oil
CAD	Charged aerosol detector
FAs	Fatty acids
RSM	Response surface methodology
TCM	Traditional Chinese Medicine
